# Reversed lipid-based nanoparticles dispersed in oil for malignant tumor treatment via intratumoral injection

**DOI:** 10.1080/10717544.2017.1330373

**Published:** 2017-05-26

**Authors:** Liao Shen, Zhen Zhang, Tao Wang, Xi Yang, Ri Huang, Dongqin Quan

**Affiliations:** Beijing Institute of Pharmacology and Toxicology, Beijing 100850, China

**Keywords:** Intratumoral delivery, hydrophilic agents, doxorubicin, sustained delivery, bioluminescent imaging

## Abstract

Intratumoral injection of anticancer drugs directly delivers chemotherapeutics to the tumor region, offering an alternative strategy for cancer treatment. However, most hydrophilic drugs spread quickly from the injection site into systemic circulation, leading to inferior antitumor activity and adverse effects in patients. Therefore, we developed novel reversed lipid-based nanoparticles (RLBN) as a nanoscale drug carrier. RLBNs differ from traditional nanoscale drug carriers in that they possess a reversed structure consisting of a polar core and lipophilic periphery, leading to excellent solubility and stability in hydrophobic liquids; therefore, hydrophilic drugs can be entrapped in RLBNs and dispersed in oil. *In vivo* studies in tumor-bearing Balb/c nude mice indicated remarkable antitumor activity of RLBN-DOX after a single injection, with effective tumor growth inhibition for at least 17 days; the inhibition rate was ∼80%. These results can be attributed to the long-term retention and sustained drug release of RLBN-DOX in the tumor region. In contrast, intratumoral injection of free DOX showed weaker antitumor activity than RLBN-DOX did, with the tumor size doubling by day 11 and tripling by day 17. Further, the initial burst of drug released from free DOX could produce detrimental systemic effects, such as weight loss. Histological analyses by TUNEL staining showed apoptosis after treatment with RLBN-DOX, whereas tumor cell viability was high in the free DOX group. Current results indicate that RLBNs show sustained delivery of hydrophilic agents to local areas resulting in therapeutic efficacy, and they may be a promising drug delivery system suitable for intratumoral chemotherapy.

## 1. Introduction

Hydrophilic anticancer drugs are widely used in the treatment of malignant tumors. The most common strategy for this kind of drug is systematic administration, such as intravenous injection (Carter & Soper, 1974; A'Hern et al., 1993). Unfortunately, this route of dosing for chemotherapeutic agents may lead to severe adverse effects such as neutropenia, cardiac toxicity, nausea and vomiting resulting from nonselective damage to both normal and tumor cells (Chari, 2008). Moreover, rapid clearance of chemotherapeutics from the blood reduces their anticancer effects (Chun et al., 2009; Al-Abd et al., 2010). For this reason, sustained therapeutic drug concentrations in tumor areas demand repeated chemotherapy in a short time, which strongly increases the associated risks. Local delivery of chemotherapeutics to tumors, especially inoperable malignant tumors, is an alternative strategy as it directly delivers the chemotherapeutics to the tumor and minimizes the nonselective distribution of drugs to normal organs and tissues (Westphal et al., 2003; De Souza et al., 2010). However, injecting hydrophilic anticancer drugs intratumorally is not a perfect solution, as the free drug tends to rapidly spread to other areas of the body through systematic circulation rather than remain at the injection site. In recent years, various attempts at using drug carriers such as liposomes, emulsions, *in situ* gels and microspheres have been made to achieve a high-targetable and prolonged antitumor effect and provide a high concentration of therapeutics to the tumor region with sustained release (Bao et al., 2006; Al-Abd et al., 2010; Ding et al., 2016; Wang et al., 2016; Zhang et al., 2016). However, particles with a small size, such as liposomes and nanoemulsions, have limited dwelling time (Nomura et al., 1998; Goins et al., 2016). Although polymer microspheres may offer a relatively sustained drug release at the injection site, preparation of this type of formulation [for instance, the water-in-oil-in-oil (w/o/o) coacervation method (Woo et al., 2001; Ruan & Feng, 2003; Freitas et al., 2005)] is a rather complicated process, and toxicity from residual solvents is a serious concern. Injectable hydrogels have drawn much attention recently because they make local drug delivery possible. However, preparation of *in situ* gels usually involves materials that are not commercially available (Jeong et al., 2002; Cao et al., 2007; Moreno et al., 2014; Yu et al., 2017), and the safety of these polymers is still unclear.

Oily formulations have drawn much attention as they are usually characterized by prolonged drug release and high biocompatibility (Lewis, 1995; Llovet & Bruix, 2003). However, most oil-based formulations featuring sustained release profiles are designed for hydrophobic drugs instead of hydrophilic drugs. Recently, we developed reversed lipid-based nanoparticles (RLBNs) dispersed in oil for intratumoral chemotherapy. Hydrophilic drugs can be entrapped in these lipophilic nanoparticles using a novel approach. Highly biocompatible lipid materials such as egg lecithin (EPC), 1,2-dipalmitoyl-sn-glycero-3-phosphoglycerol sodium salt (DPPG-Na) and medium-chain triglycerides (MCTs) were used in this system. A small unilamellar liposome (SUV) dispersion was prepared to construct bilayers of regularly arranged phospholipid molecules, and then the water was removed after mixing with drug. When MCTs were added to the system, hydrophobic forces drove the phospholipid molecules to juxtapose a layer around the polar core comprised of hydrophilic molecules. RLBNs differ from traditional nanoscale drug carriers in that they possess a reversed structure consisting of a polar core as the drug reservoir that can store the hydrophilic molecules (either small molecular drugs such as doxorubicin hydrochloride, cisplatin or macromolecules such as peptides) and a lipophilic periphery that leads to excellent solubility and stability in organic solvents and oil (The schematic of the RLBN structure is shown in the discussion section). With a continuous barrier formed by the oil phase, drug molecules are effectually constrained in the tumor area over long periods, while release of drugs from the injection site is avoided, which minimizes side effects.

To evaluate the antitumor application of a RLBN drug delivery system for local chemotherapy, RLBNs loaded with doxorubicin (RLBN-DOX) were prepared. DOX is a typical cationic anticancer drug that has wide application for cancer treatment (Lewis, 1995; Llovet & Bruix, 2003). EPC and DPPG-Na were used as film materials, and MCTs were used as the oil phase. EPC is a neutral lecithin with a polar head and two hydrophobic tails, whereas DPPG-Na is a negatively charged synthetic phospholipid. To construct lipid layers, anionic liposomes consisting of EPC and DPPG-Na were prepared as the first step. The solution was then mixed with DOX. In the water phase, DOX molecules adhered to the surface of liposomes via strong electrostatic interactions so that they would be stabilized between lipid layers during the process of lyophilization. When MCTs were added, phospholipid layers were restructured by hydrophobic forces, entrapping drug molecules and forming reversed nanoparticles in the MCTs. In this experiment, RLBN-DOX was successfully prepared as mentioned, and *in vitro* and *in vivo* evaluation of RLBN-DOX was performed using multiple approaches such as transmission electron microscopy (TEM) and a live image system for animals. Results show sustained release of drug from RLBN-DOX both *in vitro* and *in vivo* and improved anticancer effects in tumor-bearing nude mice. Further, no significant adverse reactions were found after a single dose of RLBN-DOX or empty RLBN, showing a high level of safety for the system.

## 2 Materials and methods

### 2.1 Materials

EPC and DPPG-Na were purchased from Lipoid GmbH (Ludwigshafen, Germany); MCTs were purchased from Hunan ER-KANG Pharmaceutical Co., Ltd. (Changsha, Hunan province, China). DOX hydrochloride was obtained from Zhejiang Hisun Pharmaceutical Co., Ltd. (Taizhou, Zhejiang province, China). Alexa Fluor^®^ 750 dye was purchased from Thermo Fisher Scientific Inc. RPMI 1640 medium and fetal bovine serum (FBS) were obtained from Gibco Co. (Paisley, UK). The antibiotics (100 U/mL penicillin and 100 U/mL streptomycin) and non-essential amino acids (NEAA) were purchased from Sigma-Aldrich Co. (St. Louis, MO). All other chemicals were of analytical reagent grade or better.

### 2.2 Preparation and characterization of RLBN-DOX

A three-step method was used to prepare RLBN-DOX. First, a blank liposome solution was obtained by a film-dispersion and hydration-sonication process as previously described (Zhang et al., 2013). DPPG-Na and EPC (1:4, w/w) were dissolved in 8 mL chloroform; the organic solvent was then removed by evaporation. Double distilled water (10 mL) was added to obtain the phospholipid suspension. A probe-type sonicator (200 W for 3 min in an ice-bath) was used to form empty SUVs. The SUV solution was mixed with DOX solution (2 mg/mL) and stirred for 10 min. The water was then removed by lyophilization. The freeze-drying process was as follows: freezing at −40 °C for 3 h; primary drying at −40 °C to −10 °C for 15 h; secondary drying at −10 °C to 20 °C for 15 h, then maintaining at 20 °C for 3 h. The chamber pressure was maintained at 10 Pa during the drying process. Finally, the solid mixture was dissolved into MCTs, and the oil dispersion was stirred at 25 °C until a clear RLBN-DOX dispersion was formed. The size distribution of RLBN-DOX was measured by dynamic light scattering (DLS); MCTs were used as the solvent and relative parameters were reset for the measurement (viscosity = 30 cP, refractive index = 1.4495, 20 °C). The morphology of reversed nanoparticles was studied by TEM. Specifically, RLBN-DOX was weighed and dissolved in 1.0 mL *n*-heptane; a 500-fold dilution of the dispersion was then dropped onto a square mesh grid. All procedures were operated in a dark environment and repeated three times to calculate the average values and standard deviations.

### 2.3 *In vitro* drug release

The release of DOX *in vitro* was studied using dialysis kits (30 kDa). Phosphate buffered saline (PBS, 0.1 mM) with different pH values (6.0, 6.5, 7.0 and 7.5) was used as release medium. DOX aqueous solution (0.5 mL) or RLBN-DOX (2 mg/mL DOX) was added to the dialysis bag and immersed in 50 mL medium under stirring (200 rpm, preheated to 37 ± 0.5 **°**C). Dialysate samples were withdrawn and replaced with an equal volume of fresh media at predetermined intervals, and the concentration of DOX in the medium was determined by high-performance liquid chromatography equipped with a Zorbax SB-C column (250 mm × 4.6 μm, 5 μm). The eluent was a suitable mixture of water, acetonitrile and phosphoric acid (52:48:0.68). Then, 1.44 g sodium lauryl sulfate was dissolved in 1000 mL of this solution and 2 N sodium hydroxide was used to adjust the pH to 4.0 ± 0.1. Cumulative release of DOX was calculated as follows:

$$\text{C}\text{u}\text{m}\text{u}\text{l}\text{a}\text{t}\text{i}\text{v}\text{e}\;\text{r}\text{e}\text{l}\text{e}\text{a}\text{s}\text{e}=\left(\frac{V_{0}C_{0}+V_{s}\sum_{1}^{n-1}C_{t}}{m_{0}}\right)\times 100\text{\%}$$

Where m_0_ is the total mass of drug in free DOX solution or RLBN-DOX oil dispersion, *V*_0_ is the initial volume, *V*_s_ is the sampling volume, *C*_0_ and *C_t_* are the drug concentrations, t and n are the sampling times. All assays were performed in triplicate in the absence of light.

### 2.4 *In vitro* cytotoxicity studies

The *in vitro* antitumor activity of free DOX and RLBN-DOX was determined by the WST-8 assay using the MCF-7 human breast adenocarcinoma cell line. Cells were seeded onto 96-well plates at a density of 8 × 10^3^ cells/well and allowed to adhere for 24 h. Then, the culture medium in each well was replaced by 200 μL DOX solution (0.5, 1 or 5 μg/mL DOX, diluted with culture medium) or 50 μL RLBN-DOX (containing 0.05, 0.5 or 5 μg/mL DOX) and 150 μL culture medium. After incubating for 24, 48, 72 or 96 h, all wells were washed with PBS to remove the drug; 100 μL PBS and 10 μL WST-8 reagent were then added to each well, and the plates were incubated for an additional 3 h. A Benchmark microplate reader (Bio-Rad Laboratory, Mississauga, Canada) with a 450-nm optical filter and a 650-nm reference wavelength was used to measure the absorbance of each well. Cell viability was calculated as follows:

$$Cell\;Viability=\frac{OD_{test}-OD_{blank}}{OD_{control}-OD_{blank}}*100\text{\%}$$

where OD_blank_ is the optical density of blank well (PSB and WST-8 reagent); OD_test_ is the optical density of test group; OD_control_ is the optical density of control group.

### 2.5 *In vivo* drug retention study

Alexa Fluor® 750 dye (AF750), known as a bright, near-infrared (IR) fluorescent dye with good water solubility, was used as an indicator to qualitatively study the retention profile *in vivo* with continuous observation. In this section, RLBN-DOX-AF750 was prepared by the same process as described in Section 2.2 above. Balb/c nude mice (supplied by Beijing Vital River Laboratory Animal Technology Co., Ltd., Beijing, China) bearing HepG2 tumors were used in these experiments. The HepG2 cell suspension (1.0 × 10^7^ cells in 100 μL of saline) was subcutaneously injected into the right flank of each animal. RLBN-DOX-AF750 (20 μL) or free DOX-AF750 was locally injected into tumors when the tumor volume reached ∼0.200 cm^3^, denoted as day 0, and the intensity of fluorescence was monitored by an *in vivo* imaging system (IVIS^®^ SpectrumCT, PerkinElmer^®^, Waltham, MA) up to day 9.

To quantitatively study the DOX concentration in tumors, 30 Balb/c nude mice bearing HepG2 tumors were randomly divided into a RLBN-DOX group (20 mice) and free DOX group (10 mice). RLBN-DOX (20 μL) or free DOX was intratumorally injected into tumors when their volumes reached approximately 0.200 cm^3^. At predetermined times, tumors were harvested. Each sample was immersed in 2 mL methanol and homogenized in an ice bath. Then, 8 mL acetonitrile was added to precipitate proteins, and each mixture was vortexed for 20 min at room temperature. DOX concentrations were determined by liquid chromatography tandem-mass spectrometry (LC-MS/MS). The rate of residual drug was calculated as follows: $Residual\;drug={(m}_{test}/m_{injection})*100\%$, where *m*_test_ is the mass of DOX determined by LC-MS/MS, and m_injection_ is the mass of DOX that was initially injected into the tumor on day 0. All procedures were conducted under dark conditions. The animal experimental protocols were approved by the Institutional Ethical Committee for Care and Use of Laboratory Animals of the Academy of Military Medical Sciences.

### 2.6 *In vivo* antitumor activity

Antitumor activity was evaluated in Balb/c nude mice bearing luciferase-expressing HepG2 tumors. Twenty mice were randomly divided into four groups (*n* = 5) as follows: Free DOX group, RLBN-DOX group, empty RLBN group and saline group. Treatment groups were intratumorally injected with RLBN-DOX or free DOX solution (2.5 mg/kg DOX, 20 μL) when the tumor volume reached 0.20 cm^3^, denoted as day 0. Body weights and tumor volumes were evaluated daily. Tumor volumes were calculated as follows:

$$\text{T}\text{u}\text{m}\text{o}\text{r}\;\text{V}\text{o}\text{l}\text{u}\text{m}\text{e}\;\left(mm^{3}\right)=\frac{1}{2}*\;\text{a}\;*\;{\text{b}}^{2}$$

In this equation, *a* and *b* represent the largest and smallest tumor diameters, respectively. Bioluminescent imaging (BLI) was used to monitor the tumor cell activity on days 0, 3, 6, 9, 13 and 17. Briefly, 0.2 mL d-Luciferin (15 mg/mL) was administered to tumor-bearing mice via intraperitoneal injection; after 5–10 min, mice were transferred to an *in vivo* imaging system to record tumor region bioluminescence under light anesthesia.

### 2.7 Histological analysis

On day 17, mice were euthanized and the tumors were individually harvested. The tissues were immediately immersed in 10% formalin and embedded in paraffin. The embedded specimens were sectioned and stained with hematoxylin and eosin (H&E). Apoptotic cells in tumor tissues were labeled using an *In Situ* Cell Death Detection Kit (TUNEL assay, Roche, Berlin, Germany), and the nuclei were stained with 4′, 6-diamidino-2-phenylindole (DAPI). All processes strictly followed standard operating procedures, and the slides were examined using an inverted fluorescence microscope (NIKON ECLIPSE TI-SR, Tokyo, Japan).

### 2.8 Statistical analysis

All data are presented as means ± SD. Either Student's *t*-test or a two-way analysis of variance (ANOVA) was used to evaluate the data. A *p* value less than .05 was considered to be statistically significant.

## 3. Results

### 3.1 Preparation and characterization of RLBN-DOX

**Figure 1.**
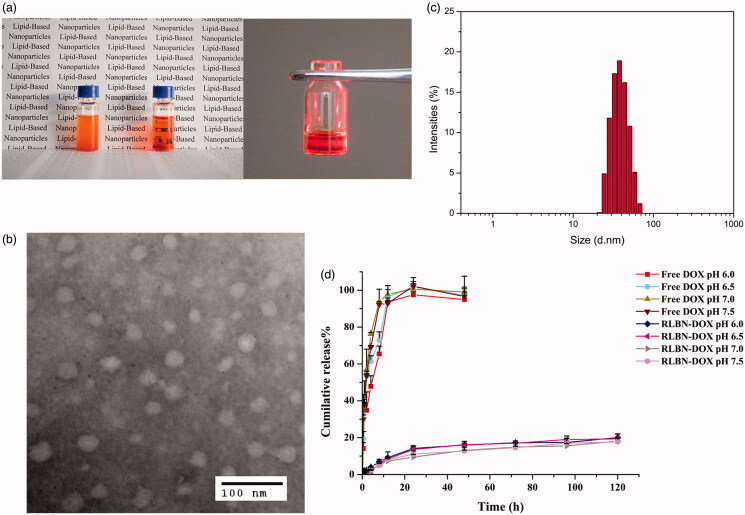
Characteristics of RLBN and RLBN-DOX. (a) Photograph of RLBN-DOX (right), simple mixture of MCT, phospholipids and DOX (left), and Tyndall effect of RLBN-DOX. (b) TEM image of RLBN-DOX (scale bar =100 nm). (c) Size distribution of RLBN measured by DLS. (d) Time curves for the in vitro release of DOX from RLBN-DOX in PBS with different pH (means ± SD, *n* = 3). RLBN: reversed lipid-based nanoparticle; MCT: medium chain triglyceride; DOX: doxorubicin; RLBN-DOX: reversed lipid-based nanoparticle loaded with doxorubicin; PBS: phosphate buffered saline; TEM: transmission electron microscopy; DLS: dynamic light scattering. SD: standard deviation.

As shown in Figure 1(a), a clear oil dispersion of RLBN-DOX was prepared using the process described in Section 2.2. TEM images of RLBN-DOX are presented in Figure 1(b). Several spherical nanoparticles were observed, and their sizes were approximately 30 to 70 nm. DLS results show a similar size distribution. RLBN-DOX was more stable and homogenous than the simple mixture containing MCT, EPC, DPPG-Na and DOX, as the large particles of phospholipids and DOX would settle from oil very quickly, suggesting superior properties for clinical application and long-term storage.

### 3.2 *In vitro* drug release

The *in vitro* cumulative release profiles of DOX from RLBN-DOX and free drug solution are shown in Figure 1(d). PBS with different pH values was used as the release medium. Approximately 100% free DOX was released from the dialysis bag within 12 h. In contrast, DOX from RLBN-DOX was released at a much slower rate in a bi-phasic pattern. A slight initial burst was observed during the first stage (0–24 h), with approximately 15% of the total DOX released from RLBN-DOX. DOX was released at a much slower rate during the second stage (5%, day 1–5). It is worth noting that the rate of DOX released from RLBN-DOX was not significantly affected by pH, indicating that release of the drug was stable under physiological conditions.

### 3.3 *In vitro* cytotoxicity studies

**Figure 2.**
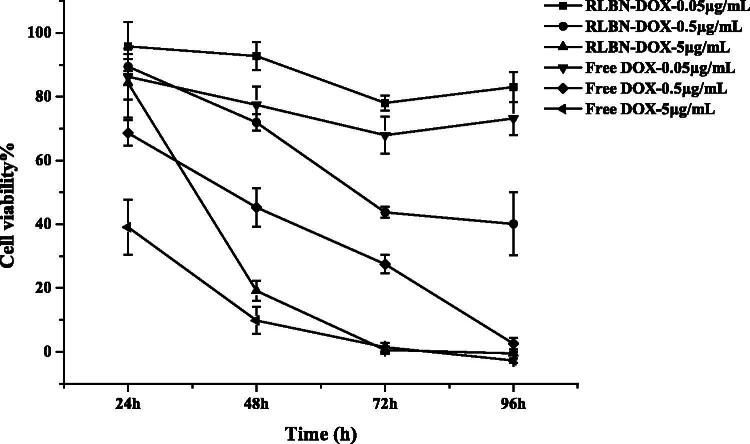
*In vitro* cytotoxicity of RLBN-DOX and free DOX solutions in MCF-7 cells (means ± SD, *n* = 5). RLBN-DOX: reversed lipid-based nanoparticle loaded with doxorubicin; DOX: doxorubicin; SD: standard deviation.

The MCF-7 cell line was used to evaluate *in vitro* cytotoxicity of free DOX and RLBN-DOX at different concentrations. Figure 2 shows inhibition rates at 24, 48, 72 and 96 h as determined by the WST-8 assay. The group receiving free DOX showed a relatively stronger cytotoxic effect within 48 h than that of the group receiving RLBN-DOX; however, after incubating for 96 h with RLBN-DOX, when a concentration of 0.05 μg/mL was used, inhibition rates reached the same level. This phenomenon might be attributable to the slow release of DOX from the oil phase. The group receiving blank RLBN showed no cytotoxic effect on MCF-7 cells (data not shown), indicating low toxicity of blank RLBN. The *in vivo* antitumor activity of RLBN-DOX is reported in *In vivo* drug retention study.

### 3.4 *In vivo* drug retention study

**Figure 3.**
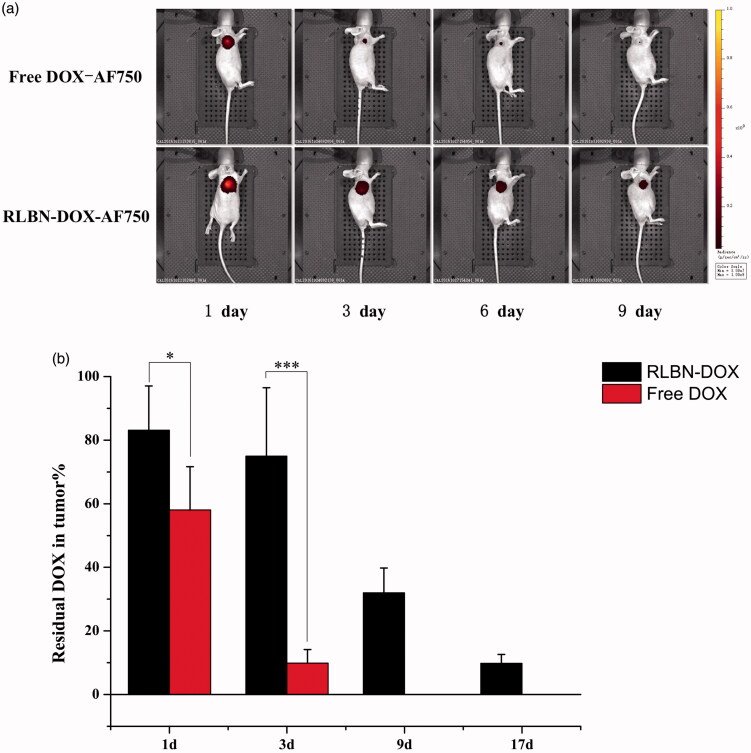
(a) Fluorescence in mice administered free DOX-AF750 or RLBN-DOX-AF750, as measured with an *in vivo* imaging system on days 1, 3, 6 and 9. Free DOX-AF750 or RLBN-DOX-AF750 was intratumorally administered on day 0. (b) Residual DOX in tumors at predetermined times (mean ± SD, *n* = 5). DOX in the Free DOX group was not detectable at day 9 and 17. DOX-AF750: doxorubicin with Alexa Fluor^®^ 750 dye (AF750); RLBN-DOX-AF750: reversed lipid-based nanoparticles loaded with doxorubicin and AF750; DOX: doxorubicin; RLBN-DOX: reversed lipid-based nanoparticle loaded with doxorubicin; SD: standard deviation. *p < .05, ***p < .001.

AF750 was used for continuous observation of the drug retention profile *in vivo*. As shown in Figure 3(a), the intensity of free DOX-AF750 decreased significantly during the first 3 days. In contrast, fluorescence was observed in the tumor region at least 9 days after a single injection of RLBN-DOX-AF750. These results indicate prolonged retention and sustained release of hydrophilic agents.

DOX in the tumors from each group was quantified, and the results are shown in Figure 3(b). After a single injection of RLBN-DOX, 83.14 ± 13.9%, 75.00 ± 21.48%, 31.97 ± 7.82% and 9.80 ± 2.81% DOX remained in tumors on days 1, 3, 9 and 17, respectively. Further, 58.07 ± 13.57% and 9.86 ± 4.3% residual DOX in tumors was found in the free DOX group on days 1 and 3, respectively. It is likely that free DOX solution did not remain in the tumor region, and approximately 40% of the drug was released from the injection site during the first day of treatment. In contrast, a mild initial drug release was measured in the RLBN-DOX group, with approximately 15% DOX released after the first day of treatment and approximately 25% released after three days. Prolonged drug retention of RLBN-DOX was also confirmed, likely providing long-lasting antitumor activity in the tumor region.

### 3.5 *In vivo* antitumor activity

**Figure 4.**
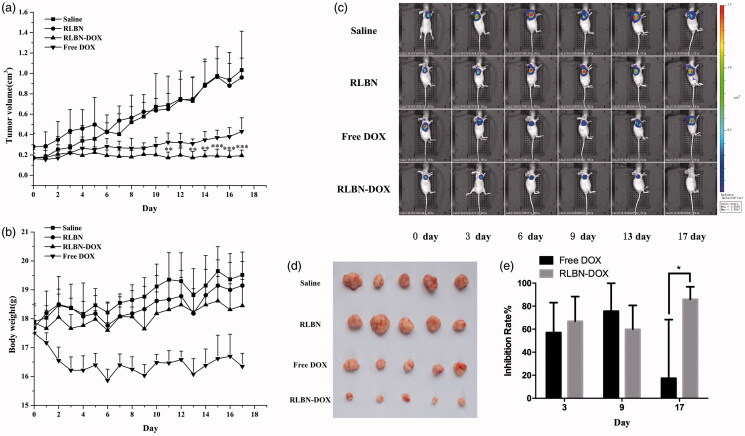
*In vivo* antitumor activity in mice bearing HepG2 cancer cell xenografts. Each group was intratumorally injected with free DOX, RLBN-DOX, empty RLBN or saline on day 0 (after recording BLI results). (a) Changes in tumor volume. Data are represented as the means ± SD (*n* = 5). *p < .05, **p < .01, ***p < .001, compared with free DOX group at corresponding time points. (b) Changes in body weight; data are given as the means ± SD (*n* = 5). (c) BLI images of mice bearing HepG2 cancer cell xenografts. (d) An image of excised tumor masses at the time of euthanasia on day 17 after treatment in HepG2 cancer cell xenograft-bearing mice. (e) Inhibition rate calculated by changes in BLI on days 3, 9 and 17. Data are represented as the means ± SD (*n* = 5). *p < .05; DOX: doxorubicin; RLBN-DOX: reversed lipid-based nanoparticles loaded with doxorubicin; BLI: bioluminescent imaging; SD: standard deviation.

Saline, empty RLBNs, free DOX and RLBN-DOX were administered intratumorally to mice bearing HepG2 xenografts on day 0. The average tumor volume of each group was calculated as shown in Figure 4(a). The tumor volumes of the Free DOX group began to increase from day 3, and the average tumor volume had doubled by day 11; by day 17, the mean tumor volume in the free DOX group was almost triple that measured on day 0 (0.165 ± 0.054 cm^3^ on day 0 versus 0.430 ± 0.135 cm^3^ on day 17). In contrast, RLBN-DOX suppressed tumor growth throughout the experimental period, with the average tumor volume on day 17 only slightly higher from that observed on day 0 (0.173 ± 0.082 cm^3^ on day 0 versus 0.194 ± 0.053 cm^3^ on day 17). The average tumor volume was 1.032 ± 0.381 cm^3^ in the saline group and 0.998 ± 0.233 cm^3^ in the empty RLBN group on day 17, indicating that empty RLBNs did not cause cytotoxicity in tumor tissues. The BLI assay, which continuously monitors tumor growth using cells specifically engineered to emit visible light (Jenkins *et al.*, 2003), showed similar results. In this experiment, a subcutaneous xeno-transplanted tumor model of firefly-luciferase-expressing HepG2 was established in nude mice. As shown in Figure 4(c), during the early period (from day 0 to day 9), free DOX had a similar tumor suppressive effect (60–80% inhibition rate, shown in Figure 4(e)) on tumor cells as that of RLBN-DOX; however, BLI intensity steadily increased in the free DOX group after 9 days of treatment. On day 17, the inhibition rate in the free DOX group decreased to approximately 20% on average, indicating significant tumor growth. In the RLBN-DOX group, inhibition of tumor growth was maintained at 60–80% on average (shown in Figure 4(e)), and, as shown in Figure 4(d), tumor size was remarkably suppressed up to day 17.

The average body weights of the mice during the 17-day experiment are shown in Figure 4(b). A sharp decline in body weight was found in the free DOX group. This phenomenon is likely attributable to the high systemic toxicity of free DOX. The body weight of mice in the RLBN-DOX group was similar to that observed in the saline and RLBN group, indicating that intratumorally injecting RLBN-DOX caused no serious damage to the mice and resulted in a high level of safety.

### 3.6 Histological analysis

**Figure 5.**
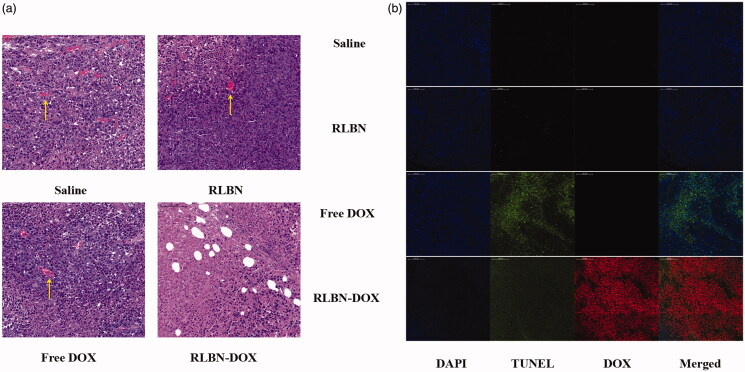
(a) H&E-stained histological sections of tumors on day 17 after intratumoral injection of saline, empty RLBN, free DOX and RLBN-DOX in xenograft-bearing mice. The yellow arrows indicate blood vessels (scale bar =100 μm). (b) DAPI staining (nuclei), TUNEL staining (apoptotic cells), red fluorescence (DOX) and merged images of tumors on day 17 after intratumoral injection of saline, empty RLBN, free DOX and RLBN-DOX in xenograft-bearing mice (scale bar =200 μm). H&E: hematoxylin and eosin; DAPI: 4',6-diamidino-2-phenylindole; TUNEL: terminal deoxynucleotidyl transferase dUTP nick end labeling; DOX: doxorubicin; RLBN: reversed lipid-based nanoparticles; RLBN-DOX: reversed lipid-based nanoparticles loaded with doxorubicin.

H&E-stained histological sections of tumors treated with saline, empty RLBN, free Dox and RLBN-DOX on day 17 after administration are shown in Figure 5(a). Blood vessels were observed in tumors injected with saline and empty RLBN, as indicated by the yellow arrows, and no significant necrosis was found in these slides. Tumors treated with free Dox showed a few necrotic regions; however, several blood vessels were found, suggesting the presence of viable tumors in this group. In contrast, tumors treated with RLBN-DOX showed much larger regions containing necrotic tissue, and no blood vessels were observed.

TUNEL staining was used for histological analyses. TUNEL-stained apoptotic cells fluoresce green and DAPI-stained nuclei in living cells fluoresce blue. As shown in Figure 5(b), only a few apoptotic tumor cells were found in the control groups injected with saline and RLBNs, suggesting that tumor cells were highly viable and that RLBNs are highly biocompatible. Some apoptotic tissues were observed in the free DOX group; however, the large area of bright blue suggests a significant number of viable tumor cells, indicating the potential reoccurrence of the neoplasm. In contrast, significant apoptosis was observed in the group treated with RLBN-DOX, and tumor cell viability was significantly suppressed, as shown by DAPI staining. The large area of red fluorescence is a result of residual DOX in the tumor region, suggesting prolonged retention of our formulation.

## 4. Discussion

Chemotherapy is a primary approach to the fight against cancer. Patients with malignant tumors are more likely to be treated with systematic strategies such as intravenous administration of anticancer therapeutics. Repeated mega-dosing of drugs is required to achieve a therapeutic concentration that kills tumor cells. However, the clinical use of chemotherapeutics is severely restricted by dose-limiting side effects resulting from nonspecific toxicity to normal cells. Local chemotherapy has drawn extensive attention as it delivers drugs directly to the tumor region. In this study, we developed an RLBN drug delivery system for local administration of hydrophilic anticancer therapeutics. Highly biocompatible lipid materials such as EPC, DPPG-Na and MCTs were used in this system. The phospholipid molecule features two hydrophobic fatty acid “tails” and a hydrophilic “head” in its structure. Because of these unique characteristics, a lamellar structure is formed in both water and organic solvents. Furthermore, the phospholipid layer features mobility and flexibility that can be restructured under certain conditions. Therefore, we prepared an SUV dispersion to construct bilayers of regularly arranged phospholipid molecules and then removed the water after mixing with drug. During this process, DPPG-Na became negatively charged, enabling the DOX molecules to adhere to the lipid layers through strong electrostatic interactions.

**Figure 6.**
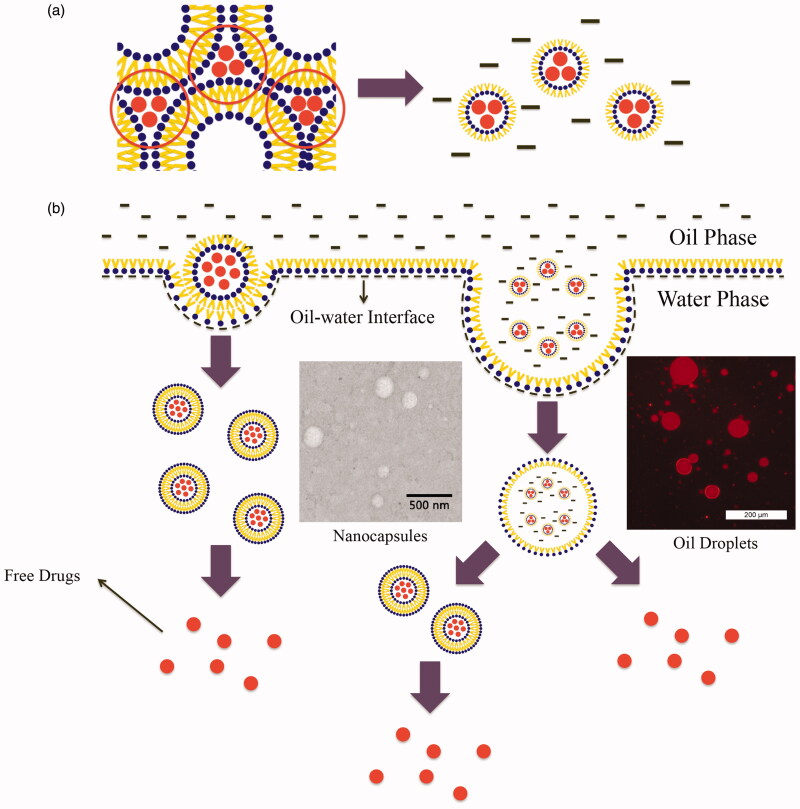
(a) Schematic of RLBN-DOX formation. The red dots indicate DOX molecules, black bars indicate MCTs. (b) Schematic representation of RLBN-DOX behavior on the oil/water (o/w) interface. TEM images of nanocapsules in release medium (scale bar =500 nm) and inverted fluorescence microscope images of oil droplets are shown (scale bar =200 μm); the red fluorescence is from DOX. MCT, medium chain triglycerides; RLBN-DOX, reversed lipid-based nanoparticles loaded with doxorubicin; TEM, transmission electron microscopy.

When MCTs were added to the system, hydrophobic forces drove the phospholipid molecules to juxtapose a layer around the polar core comprised of hydrophilic molecules. The head groups of the phospholipid molecule were positioned to the interior, and the fatty tails were arranged outward so that the hydrophilic drug could be entrapped into the nanoparticles and dispersed in oil. The process is shown in Figure 6(a).

DOX was used as a model hydrophilic anticancer agent, and RLBN-DOX was prepared as described in Section 2.2. On the microscale, numerous spherical nanoparticles were found in the oil phase using TEM and DLS, and the diameters of these particles ranged from 30 to 70 nm. Further, hydrophilic molecules were entrapped in the hydrophobic nanoparticles and homogenously dispersed into the oil phase as a clear dispersion. Neither toxic organic solvents nor extreme conditions were involved in the preparation, suggesting that RLBNs can be further used as a vehicle for delivery of therapeutics with poor stability, such as protein and DNA.

Results of the *in vitro* drug release studies using RLBN-DOX show a significantly sustained pattern, with nearly 80% of the DOX restrained in the oil phase after 5 days. Oil and water are immiscible under normal circumstances; therefore, crossing of the o/w interface is a very slow process because the oil phase represents a continuous barrier. In addition, amphipathic phospholipids with a polar head and two hydrophobic tails can self-assemble, forming closed lipid-capsules that might entrap oil droplets containing reversed nanoparticles when dispersed in water, which were detectable in the water phase. As such, we mixed RLBN-DOX and PBS (1:9, v/v), and collected the water phase after two days stirring (100 rpm); nanoparticles were then detected by TEM, and oil droplets were observed by inverted fluorescence microscopy, showing that the red color came from DOX. The behavior of RLBN-DOX on the o/w interface is shown in Figure 6(b).

The MCF-7 cell line was used to evaluate antitumor activity *in vitro*. A delayed cytotoxic effect was observed in the RLBN-DOX group. This phenomenon may be attributable to the slow release of nanoparticles and a different uptake mechanism.

A live image system was used to observe the drug retention profile *in vivo* after a single injection of RLBN-DOX-AF750. Alexa Fluor 750 is a near-IR fluorescent dye with good water solubility that allows for visualization of hydrophilic agents after intratumoral injection. A sharp decrease in the fluorescence of free DOX-AF750 was observed during the first 3 days, whereas a strong RLBN-DOX-AF750 fluorescent signal was detected up to day 9. These results indicate that RLBNs effectively prolonged the retention of hydrophilic agents within the tumor site after intratumoral injection.

BLI was used to monitor antitumor activity in mice. Free DOX resulted in limited cytotoxicity and a sharp decrease in body weight (∼9% weight loss). In contrast, RLBN-DOX provided long-lasting suppression of tumor growth with less weight loss in mice. This may be explained by the pharmacokinetics of DOX at the tumor site. In the free DOX group, DOX aqueous solution was directly injected into the tumor, with obvious cytotoxicity observed in the tumor tissues within a short period; however, approximately 90% of the DOX was released from the tumor site into the systemic circulation within 3 days. This led to obvious side effects and inferior antitumor activity. The low residual DOX also led to regrowth of tumor cells, as shown by the continually increasing BLI signal in tumors from the free DOX group after day 9. However, high concentrations of DOX were maintained within the tumor site in the RLBN-DOX group, providing prolonged inhibition of tumor growth.

No apoptotic cells were observed after injection with saline or RLBNs during the experimental period, as shown by TUNEL assay. Tumor growth was inhibited by local administration of RLBN-DOX and DOX. However, a single administration of free DOX did not sustain the antitumor activity for the entire experimental period, and numerous tumor cells survived; this is an extremely undesirable effect as it tremendously raises the risk of multi-drug resistance (MDR) in clinical practice. In contrast, DOX was detectable in the RLBN-DOX group on day 17, indicating a much longer antitumor effect that may effectively avoid MDR.

## Conclusions

In this study, a novel RLBN drug delivery system loaded with a hydrophilic drug was developed for intratumoral chemotherapy. DOX, a common hydrophilic anticancer agent, was used as the model drug. RLBN-DOX manifested as a clear and homogenous oil dispersion. Numerous reversed nanoparticles were characterized by TEM and DLS, and the size distribution was from 30 to 70 nm. Results show that RLBN-DOX effectively prolonged the retention of drugs and avoided the initial burst of drug release, thus providing sustained suppression of tumor growth for 17 days and fewer side effects in tumor-bearing mice. Although further studies will be required to determine the mechanisms of drug release and the uptake process of tumor cells, the present findings show that RLBNs have potential as a local chemotherapy delivery system.
